# The Oligomeric State of Vasorin in the Plasma Membrane Measured Non-Invasively by Quantitative Fluorescence Fluctuation Spectroscopy

**DOI:** 10.3390/ijms25074115

**Published:** 2024-04-08

**Authors:** Junyi Liang, Adam W. Smith

**Affiliations:** 1 Department of Chemistry, University of Akron, Akron, OH 44325, USA; 2 Genomic Medicine Institute, Cleveland Clinic, Cleveland, OH 44195, USA; 3 Department of Chemistry and Biochemistry, Texas Tech University, Lubbock, TX 79409, USA

**Keywords:** vasorin (VASN), fluorescent proteins, fluorescence fluctuation spectroscopy, membrane protein multimerization

## Abstract

Vasorin (VASN), a transmembrane protein heavily expressed in endothelial cells, has garnered recent interest due to its key role in vascular development and pathology. The oligomeric state of VASN is a crucial piece of knowledge given that receptor clustering is a frequent regulatory mechanism in downstream signaling activation and amplification. However, documentation of VASN oligomerization is currently absent. In this brief report, we describe the measurement of VASN oligomerization in its native membranous environment, leveraging a class of fluorescence fluctuation spectroscopy. Our investigation revealed that the majority of VASN resides in a monomeric state, while a minority of VASN forms homodimers in the cellular membrane. This result raises the intriguing possibility that ligand-independent clustering of VASN may play a role in transforming growth factor signaling.

## 1. Introduction

**Figure 1:**
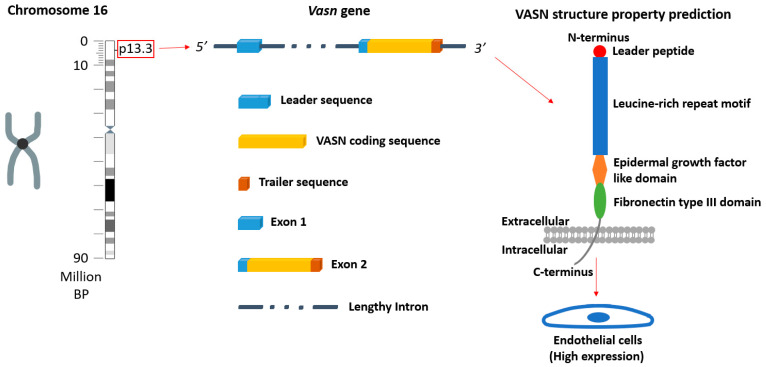
The location of the *vasn* gene in a human chromosome (chromosome 16), the structural properties of the gene, and a prediction of the VASN protein structure (predicted by RaptorX http://raptorx.uchicago.edu (accessed on 2 April 2024)) [15,16].

Vasorin (VASN) is a single-pass membrane protein comprising 673 residues [1]. In the species of *Homo sapiens* and *Mus musculus*, chromosome 16 is home to *vasn*, the gene encoding VASN. *Vasn* consists of two exons separated by a considerably large intron (Figure 1). Notably, both the gene and the protein exhibit a high degree of conservation from species to species. This remarkable conservation implies a conserved role of VASN during evolution. Specific expression patterns of VASN vary depending on the developmental stage and physiological conditions. However, a consensus has emerged that VASN is highly expressed in smooth muscle cells and endothelial cells, the essential cell types in blood vessels [2,3]. Lately, VASN has been shown to play a key role in vascular development and homeostasis [4,5]. Yet, a mechanistic understanding is not currently available. A key unanswered question is the oligomeric state of VASN in its native membranous environment. This is important because receptor oligomerization is a recognized mechanism for modulating the intensity of signal transduction [6,7,8]. For instance, many receptors in the seven-pass G protein-coupled receptor (GPCR) class-C family turn to receptor clustering to enhance the extent of downstream signaling events [9,10,11,12], and pre-formed homodimeric receptors are required for some receptors to be activated [13,14]. We were motivated to characterize the oligomeric state of VASN in the plasma membrane and to discover if any preformed, ligand-independent clustering was present.

The characterization of the homo-oligomeric state of VASN in the membrane needs to meet two requirements, in our opinion. Foremost, the measurement needs to be performed in live cells with low toxicity. Additionally, the measurement should be quantitative. In the present study, we chose to use fluorescence fluctuation spectroscopy. In doing so, we chose to label VASN with fluorescent proteins, which are biocompatible owing to the genetic encodability of fluorescent proteins [17,18]. Several fluorescence methods can be employed to measure membrane protein oligomerization [19,20]. For example, bimolecular fluorescence complementation (BiFC) [21] and fluorescence resonance energy transfer (FRET) [22] are commonly employed, but they can be challenging to calibrate and do not directly resolve the protein concentration and diffusion [21,22]. Instead, the fluorescence method we selected to quantify VASN’s biophysical properties was fluorescence fluctuation spectroscopy (FFS) [23,24].

FFS is based on the fundamental idea that the fluorescence intensity changes within a diffraction-limited volume (3D) or area (2D) are caused by fluorophores diffusing freely into and out of this region over time. The fluctuating signal may be used to accurately quantify the biophysical properties of the fluorescently tagged biomacromolecules [25,26,27,28], such as a cross-correlation between the dual fluorescence emitted from the tagged membrane green fluorescent proteins (GFPs) and red fluorescent proteins (RFPs) moving into and out of the tiny confocal area. G(𝝉) is a parameter of the signal’s self-resemblance with the passage of time. G_g_ and G_r_ are the autocorrelations of GFP and RFP, while G_x_ is the cross-correlation. F_g_ and F_r_ stand for the detected fluorescence fluctuation at a certain moment for GFP and RFP, respectively.

G(𝝉) = <F_g_(t)F_r_(t + 𝝉)>/<F_g_(t)F_r_(t)> − 1; f_c_ = max (G_x_(0)·G_r_(0)^−1^, G_x_(0)·G_g_(0)^−1^)

G(𝝉) may also be fitted with a two-dimensional Brownian motion model, as is in the case of membrane proteins, to yield the number of GFP- and RFP-tagged membrane proteins (for soluble proteins, the fitting equation should be a 3D diffusion model). The fraction correlated (f_c_) is calculated by determining the maximum amplitude of the cross-correlation curve and is the figure of merit for rating the degree of homo-oligomerization.

## 2. Results

**Figure 2:**
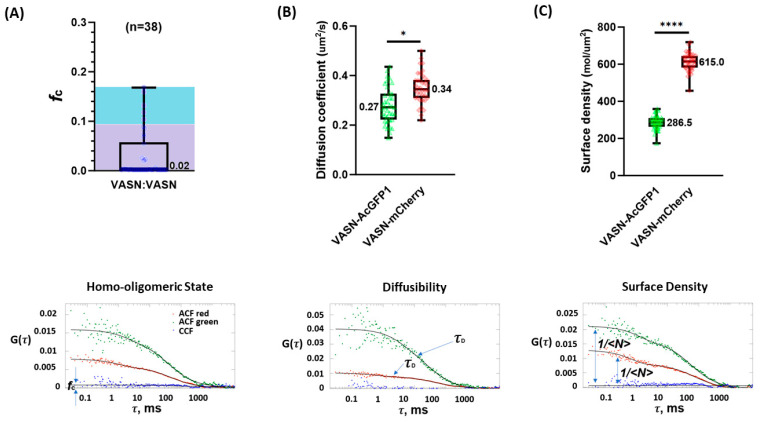
The biophysical characterization of VASN in the cellular membrane including its oligomeric state (* and **** are markers denoting slight and notable statistical significance). (**A**) The lower panel is a representative set of single-cell PIE-FCCS data. The fraction correlated (f_c_) can be obtained from the amplitude of the CCF as the CCF is dependent on the distribution of monomers, dimers, trimers, and multimers. The upper panel is VASN’s *f_c_* as measured from 38 cells, which clearly established that the vast majority of VASN resided in the membrane in a monomeric state. (**B**) The mobility of VASN-AcGFP1 and VASN-mCherry, as denoted as the diffusion coefficient in the (**upper panel**), was computed with the information of the diffusers’ average dwell time available in the measurement curve (**lower panel**). (**C**) ACF’s amplitude is an indicator of the average number of diffusers as these two are in an inverse proportional relationship.

In previous research, our group calibrated the expected *f_c_* values for monomeric and dimeric receptors [29], which we used to interpret the VASN results. Cells expressing primarily dimers were expected to have *f_c_* values in the range of 0.094 to 0.170 [29]. Among the 38 VASN-AcGFP1- and VASN-mCherry-expressing COS-7 cells we measured by FCCS, 33 yielded an *f_c_* value below the dimer range, representing 86.8% of the population. The remaining cells yielded an *f_c_* value in the dimer range, representing 13.2% of the population (Figure 2A). These FCCS results clearly indicate that the majority of VASN resides in the plasma membrane in a monomeric state, and that the VASN existing as dimers is only a small minority.

It should also be noted that the mobility of VASN-AcGFP1 and VASN-mCherry are relatively close, as shown in Figure 2B. The molecular mobility is to a large extent dependent on the molecular mass. AcGFP1 and mCherry, both noted for their characteristic 11 beta sheet barrel structure, are minimally different in weight. The diffusion coefficients for VASN-AcGFP1 and VASN-mCherry were relatively similar. It may be that unlabeled VASN will be more mobile than shown here because of the AcGFP1 and mCherry domains. 

Our attention now turns from mobility to the surface molecular density. In spite of the equal molar amounts of DNA encoding transfected VASN-AcGFP1 and VASN-mCherry, as ensured by our single-plasmid-based dual-expression architectural design, the overall molecular density of VASN-mCherry is notably higher than that of VASN-AcGFP1 (Figure 2C). This might be due to the differences in the transcription and translational efficiencies of these two DNAs. But the density of hundreds of VASN per square micron is in line with the density of VASN in certain physiological conditions, such as in glioma, as VASN is reported to be overexpressed in certain tumors, including brain tumors [30]. Note that the absolute values of the molecular densities of VASN-AcGFP1 and VASN-mCherry were obtained after curve fitting the three correlations, two ACFs, and one CCF. The routine calibration focused on aligning the optical pathway to the point where the instrument reflected the near-perfect cross-correlation of a standard DNA chain labeled with both green and red dyes (3D diffusion in solution) [29], so that the *f_c_* value, and by extension, the oligomeric state quantification we cared most about, was precise. 

## 3. Discussion

We applied a class of fluorescence fluctuation spectroscopy, PIE-FCCS, to investigate the homo-oligomeric state of vasorin, a key membrane protein involved in vascular development and maintenance. Using PIE-FCCS, our study clearly determined that the vast majority of VASN resides in the cellular membrane in a monomeric state. A legitimate concern is that the utilization of fluorescent protein tags will impact the oligomerization capacity of a membrane protein, due to the potential steric hindrance brought about by FP’s sheer size. We do not think this possibility can be categorically ruled out. But the impact of the fluorescent protein tags on the oligomeric state of the membrane protein is minimal. A case in point is the evaluation of mGluR2, a known constitutive dimeric receptor [31], by FCCS. Measurement of mGluR2 tagged with a fluorescent protein or tagged chemically (small-sized chemical dyes: ATTO488-BG and DY549P1-BG) yielded close f_c_ values, both identified as dimers [32]. Hence, PIE-FCCS has been invaluable for use in evaluating the oligomeric state of membrane proteins.

This finding concerning monomeric VASN contrasts sharply with certain key receptors, such as the erythropoietin receptor also in the vascular system [33] and neurotrophic factor receptor TrkB [34], which are known to exist in the membrane as pre-formed dimers conducive to rapid receptor clustering and activation. This major difference indicates that VASN may go through an alternative pathway for receptor activation, potentially through heterocomplex pre-formation. One other possibility is that a VASN monomer is a functional unit by itself, without a need to associate with other VASN monomers in carrying out its function. 

The two potential VASN activation pathways as indicated by its monomeric state in the membrane have their own implications for the ongoing therapeutic strategy of targeting VASN, be it in a therapy for glioma or one for other vascular-related conditions. Should VASN activate via hetero-interaction with another key receptor, it will be imperative to first identify this key and yet unknown receptor. The subsequent therapeutic development may then focus on screening compounds that may enhance or weaken this hetero-interaction, depending on whether activation or deactivation of VASN is the desired outcome. Alternatively, should a VASN monomer be functional as a single unit, the investigative focal point will then be to understand the specific structural change in VASN that corresponds to its activation. Then, the therapeutic development should involve designing the compounds to turn VASN on or off by modulating VASN’s conformation. In essence, our discovery of VASN’s tendency of residing in the plasma membrane as a monomer answers the important question of what oligomeric state VASN is in, but it also raises more questions about its activation mechanism and therapeutic implications.

## 4. Methods and Materials

### 4.1. Pulsed Interleaved Excitation Fluorescence Cross-Correlation Spectroscopy (PIE-FCCS) and Experimental Description 

**Figure 3:**
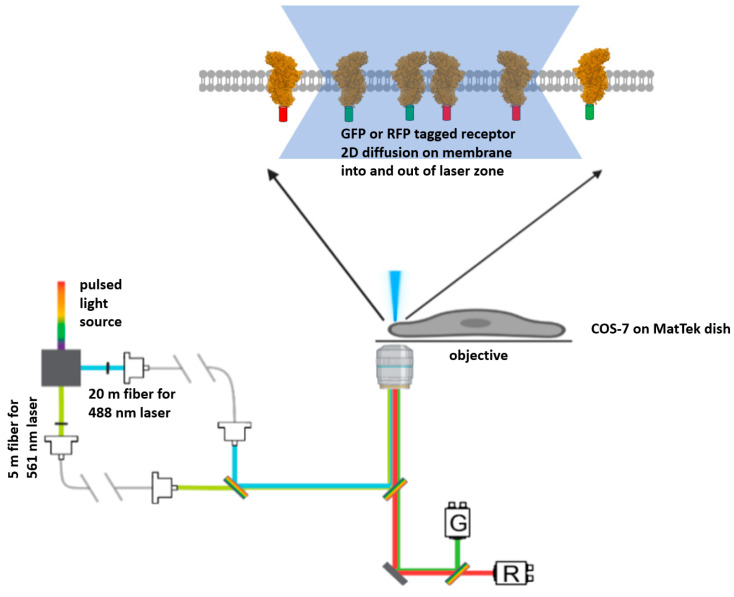
The instrumental set up: Simplified schematic of the PIE-FCCS optical path. A pulsed light source is used to generate two laser beams for excitation. The beams pass through optical fibers with a length difference of 15 m to hard write a 50 ns difference in excitation time. (Note that 50 ns is roughly an order of magnitude longer than AcGFP1 (GFP) and that mCherry (RFP) needs to decay back to the ground state from the excited state.) Fluorescent tagged membrane proteins (a general receptor shown here in this figure) would diffuse into and out of the confocal area under laser excitation, resulting in fluctuations in fluorescence intensity captured by photon counting.

A commercially available inverted confocal microscope (Nikon, Tokyo, Japan) was custom built to perform pulsed interleaved excitation fluorescence cross-correlation spectroscopy (PIE-FCCS) [35,36]. A white laser pulse from a supercontinuum fiber laser (repetition rate of 10 mHz) was utilized as the excitation light source. The separation of the two beams was realized as shown in the optical pathway to generate 488 nm and 561 nm laser beams. With this white laser split, these two excitation beams travelled through two separate optical fibers. These two optical fibers had a length difference of 15 m (Figure 3). Temporally, a 50 ns delay was introduced in this way, which enabled the removal of spectral crosstalk, and this design also allowed AcGFP1 and mCherry to decay back to the ground state before the excitation by the next wave of laser pulse. Spatially, the 488 and 561 nm laser beams were meticulously aligned to excite the same confocal spot on the upper and lower cellular membranes. Hence, the data were always collected around the edges of the cells, which also minimized the excitation of internalized VASN-AcGFP1 and VASN-mCherry that were diffusing intracellularly between the upper and lower cellular membranes.

### 4.2. Construct Architecture

**Figure 4:**
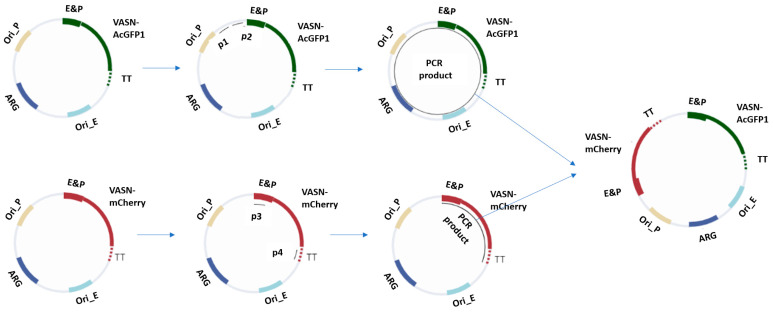
Design strategy for the single-plasmid-based, dual-expression construct. The two regular expression plasmids are the starting material. For one plasmid, nearly the entirety was amplified out with primer 1 (p1) and primer 2 (p2), including the gene encoding VASN-AcGFP1 and the regulatory units (enhancer and promoter (E&P) with transcription terminator (TT)) and survival units (origin of replication in prokaryote (Ori_P) and in eukaryote (Ori_E) and antibiotic resistance gene (ARG)). For the other plasmid, the gene encoding VASN-mCherry and the regulatory units were amplified out with primer 3 (p3) and primer 4 (p4). The fitting sticky ends were supplied onto the end of the linearized DNA in the PCR. The linear PCR products were purified and digested. The digestive products were then purified and ligated into a single circular DNA. This circularized DNA is the single plasmid for the dual expression construct.

For the exogenous expression of VASN-AcGFP1 and VASN-mCherry, we set out to adopt a novel single-plasmid-based dual protein expression over the conventional dual-expression plasmids. This single plasmid expressing both VASN-AcGFP1 and VASN-mCherry simplifies the transfection procedure and reduces labor. The starting point for the fabrication of the single plasmid for dual expression is with two regular expression plasmids, one for VASN-AcGFP1 and one for VASN-mCherry. For the regular expression plasmid for VASN-AcGFP1, nearly the entirety of the plasmid was amplified out with primer 1 (p1) and primer 2 (p2), including the VASN-AcGFP1 gene and all the regulatory and survival components, e.g., enhancer and antibiotic resistance gene. For the other regular expression plasmid for VASN-mCherry, the VASN-mCherry gene together with its regulatory components were amplified out with primer 3 (p3) and primer 4 (p4). The coupled sticky ends were fitted onto the end of the linearized DNA in the PCR process. DNA ligation was carried out to fabricate the single plasmid for the dual expression of VASN-AcGFP1 and VASN-mCherry after DNA cleanup. This procedure is graphically shown in Figure 4.

### 4.3. COS-7 Culturing and Transfection

COS-7 was stocked in a −80 °C refrigerator and promptly thawed in a water bath after being taken out. COS-7 was seeded into a 10 cm dish containing DMEM supplemented with 10% FBS and 1% Pen-Strep. The cells were then maintained in a controlled incubator at 37 °C with a 5% CO_2_ atmosphere. Subsequently, trypsin-EDTA was used as the cell dissociation agent for subculturing when COS-7 reached 80–90% confluency. The detached COS-7 was then re-seeded in fresh DMEM for sustained cell culturing. COS-7 was subcultured onto MatTek (BICO group, Gothenburg, Sweden) dishes shortly before the imaging session, and the total volume of the culturing medium was set at 500 μL. Then, 1 μg of single plasmid for dual expression was combined with 2 μL of lipofectamine 2000 in 100 μL of Opti-MEM reduced serum medium. Following a 10 min incubation at room temperature, the DNA–lipid mixture was introduced into the COS-7 culturing medium. 

### 4.4. FCCS Data Gathering and Processing

COS-7 imaging dishes were placed in a temperature-controlled (37 °C) chamber. The cells were imaged in epifluorescence mode to observe the expression and cellular localization of AcGFP1- and mCherry-labeled proteins. Upon finding a fluorescent cell, the 488 nm and 561 nm laser beams were then directed to the edge of that cell. Then, time-tagged time-resolved data (TTTR) were collected on a PicoHarp 300 (PicoQuant, Berlin, Germany). Each acquisition lasted for 10 s, with each measurement consisting of 6 acquisitions. Data were binned at 0.1 s. Time gating for mCherry was set at between 2350 and 3100, and for AcGFP1, it was set at between 1 and 600. mCherry autocorrelation, AcGFP1 autocorrelation, and mCherry-AcGFP1 cross-correlation functions were then used on the data to produce fitting curves for each acquisition. Individual datasets were then averaged and fitted with a 2D diffusion model. The fraction correlated (f_c_) was calculated from the fitted values.

## Data Availability

Raw data are available upon reasonable request.
